# Somatic mutations in homologous recombination pathway predict favourable prognosis after immunotherapy across multiple cancer types

**DOI:** 10.1002/ctm2.619

**Published:** 2021-12-19

**Authors:** Zaoqu Liu, Chunguang Guo, Jing Li, Hui Xu, Taoyuan Lu, Libo Wang, Long Liu, Xinwei Han

**Affiliations:** ^1^ Department of Interventional Radiology The First Affiliated Hospital of Zhengzhou University Zhengzhou China; ^2^ Interventional Institute of Zhengzhou University Zhengzhou China; ^3^ Interventional Treatment and Clinical Research Center of Henan Province Zhengzhou China; ^4^ Department of Endovascular Surgery The First Affiliated Hospital of Zhengzhou University Zhengzhou China; ^5^ Department of Cerebrovascular Disease Zhengzhou University People's Hospital Zhengzhou China; ^6^ Department of Hepatobiliary and Pancreatic Surgery The First Affiliated Hospital of Zhengzhou University Zhengzhou China

Dear Editor,

Homologous recombination (HR) pathway was recently implicated in modifying the antitumour response and thus might serve as a biomarker of immune checkpoint inhibitor (ICI) treatment.^1^ Thus, this study aims to comprehensively explore the clinical and molecular significance of HR mutations in ICI treatment across multiple cancer types.

The details of data and methods were descripted in Table S1 and supplementary material. HR mutations were defined as any non‐silent mutations in 17 recommended genes, including *ATM*, *BAP1*, *BARD1*, *BLM*, *BRCA1*, *BRCA2*, *BRIP1*, *CHEK2*, *ABRAXAS1*, *FANCA*, *FANCC*, *NBN*, *PALB2*, *RAD50*, *RAD51*, *RAD51C* and *RTEL1*.^2^, ^3^ According to the presence or absence of mutations in HR genes, patients were divided into HR‐Mut and HR‐wild type (WT) subgroups. The proportion of HR‐Mut cases in different cancer types was summarized in Figure 1A. To further investigate the impacts of HR mutations on ICI‐treated patients, we performed the Kaplan–Meier analysis. In memorial sloan kettering (MSK) ICI pan‐cancer cohort,^4^ patients with the HR‐Mut phenotype displayed significantly prolonged overall survival (OS) (Figure 1B). However, there was no survival difference between two phenotypes in melanoma, renal cell carcinoma, esophagogastric cancer, head and neck cancer, breast cancer, glioma and cancer of unknown primary (CUP) (Figure 1C‐I). On the other hand, the HR‐Mut phenotype showed dramatically improved OS in bladder cancer (BLCA), colorectal cancer (CRC) and non‐small‐cell lung cancer (NSCLC) (Figure 1J‐L). We also enrolled two NSCLC ICI cohorts and observed the same results, patients with HR‐Mut have better progression‐free survival and higher durable clinical benefit (Figure 1M‐N and Figure S1A,B). In multivariate Cox analysis, the HR phenotypes remained an independent protective factor for ICI‐treated patients in BLCA, CRC and NSCLC (Figure 1O‐Q). To further examine the impacts of HR mutations on patients with traditional treatments in BLCA, CRC and NSCLC, we enrolled other 11 non‐ICI cohorts. Interestingly, there was no survival difference between two phenotypes in most cohorts (Figure 2A‐I). Also, patients with the HR‐Mut phenotype had relatively lower but not significant OS in Chen‐lung adenocarcinoma (Figure 2J), and significantly lower in thoracic PDX (MSK, provisional) (PDX)‐NSCLC (Figure 2K), which were opposite to the above results in ICI cohorts. Thus, HR mutations are indicative of favourable prognosis in BLCA, CRC and NSCLC treated with ICI instead of non‐ICI.

**FIGURE 1:**
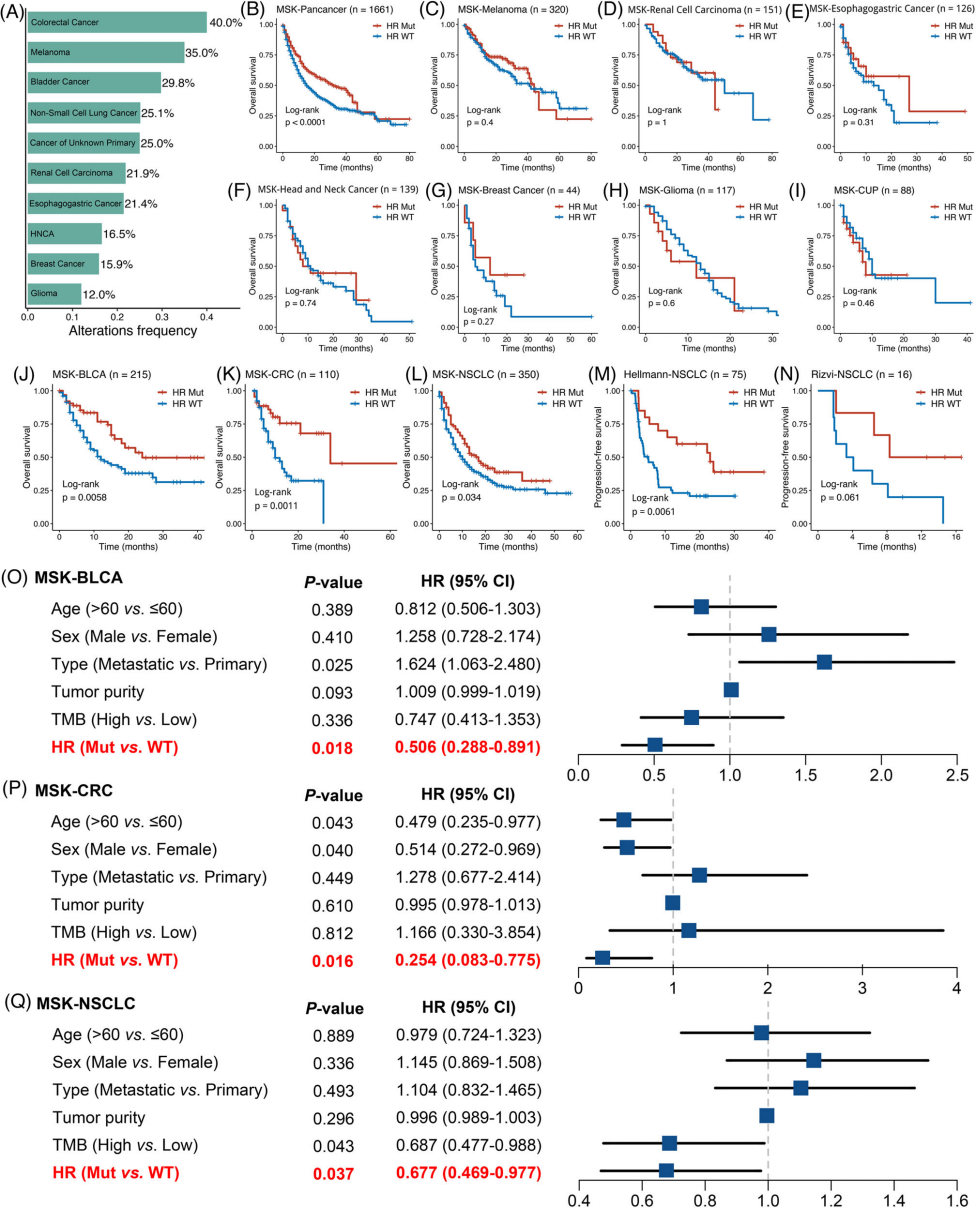
The incidence and survival analysis of homologous recombination (HR) mutations in immune checkpoint inhibitor (ICI) cohorts across multiple cancer types. (A) Frequency of HR mutations in patients with different cancer types. (B‐L) Kaplan–Meier survival curves of overall survival (OS) comparing the HR‐Mut and HR‐WT phenotypes in ICI‐treated pan‐cancer (B), melanoma (C), renal cell carcinoma (D), esophagogastric cancer (E), head and neck cancer (F), breast cancer (G), glioma (H), cancer of unknown primary (CUP) (I), bladder cancer (BLCA) (J), colorectal cancer (CRC) (K), and non‐small‐cell lung cancer (NSCLC) (L) from MSK ICI cohort. (M and N) Kaplan–Meier survival curves of progression‐free survival comparing the HR‐Mut and HR‐WT phenotypes in Hellmann‐HSCLC (M) and Rizvi‐NSCLC (N) cohorts. (O‐Q) Multivariable Cox analysis of the HR phenotypes in MSK‐BLCA (O), MSK‐CRC (P) and MSK‐NSCLC (Q)

**FIGURE 2:**
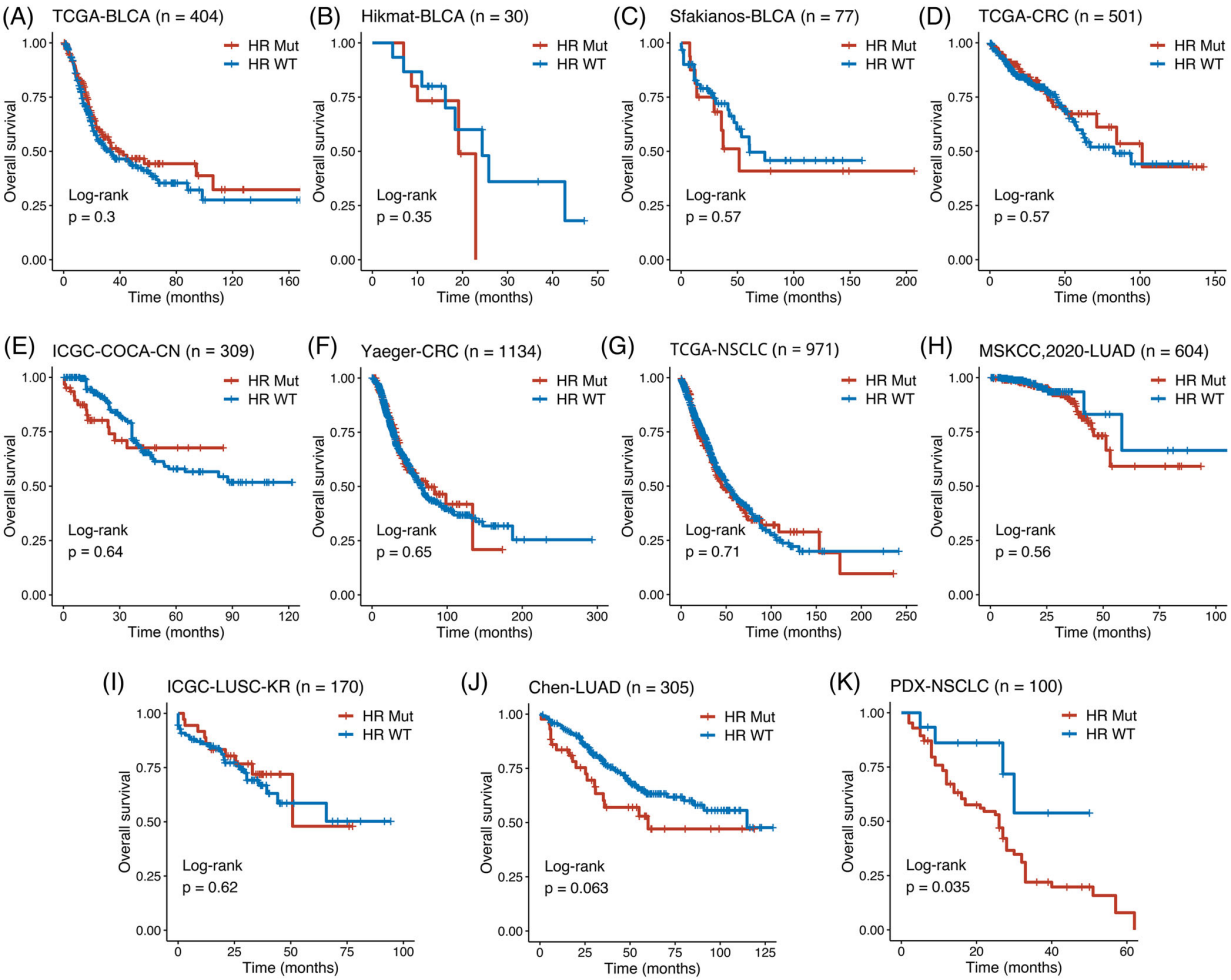
Survival analysis of homologous recombination (HR) mutations in non‐immune checkpoint inhibitor (ICI) treated bladder cancer (BLCA), colorectal cancer (CRC) and non‐small‐cell lung cancer (NSCLC). (A‐K) Kaplan–Meier survival curves of overall survival (OS) comparing the HR‐Mut and HR‐WT phenotypes in the cancer genome atlas (TCGA)‐BLCA (A), Hitmat‐BLCA (B), Sfakianos‐BLCA (C), TCGA‐CRC (D), ICGC‐COCA‐CN (E), Yaeger‐CRC (F), TCGA‐NSCLC (G), MSKCC‐2020‐LUAD (H), ICGC‐LUSC‐KR (I), Chen‐LUAD (J), PDX‐NSCLC (K)

By analysing the DNA sequencing data from MSK and TCGA pan‐cancer cohorts, we found the HR‐Mut phenotype had predominantly more single‐nucleotide polymorphisms, insertion or deletion and tumour mutation burden (TMB) (Figure 3A,B). The proportion of TMB‐high in the HR‐Mut phenotype was also substantially higher (Figure 3C‐J). Receiver operating characteristic curve (ROC) analysis revealed that HR mutations could accurately predict TMB‐high (Figure 3K‐N and Figure S2A‐D). Using the neoantigens data from 5935 patients in TCGA dataset,^5^ we further explored the association between HR mutations and neoantigen load (NAL). We found patients with HR Mut possessed dramatically more NAL (Figure S3), and the proportion of NAL‐high was also remarkably higher in the HR‐Mut phenotype (Figure S4A‐D). ROC analysis showed that HR phenotype could accurately assess tumour NAL (Figure S4E‐H). Given the latent interactions between HR pathway and mismatch repair (MMR) pathway,^6^ we further revealed the association between two DNA damage repair pathways in different cancer types.^7^ As showed in Figure S5A, there were significant HR/MMR comutation patterns in tumours. Therefore, we speculated that HR mutations may have a potential connection with microsatellite instability (MSI). Indeed, nearly all MSI‐H tumours belonged to the HR‐Mut phenotype (Figure S5B‐E). Further ROC analysis revealed that the HR mutations also could accurately predict the MSI‐H phenotype (Figure S5F‐I).

**FIGURE 3:**
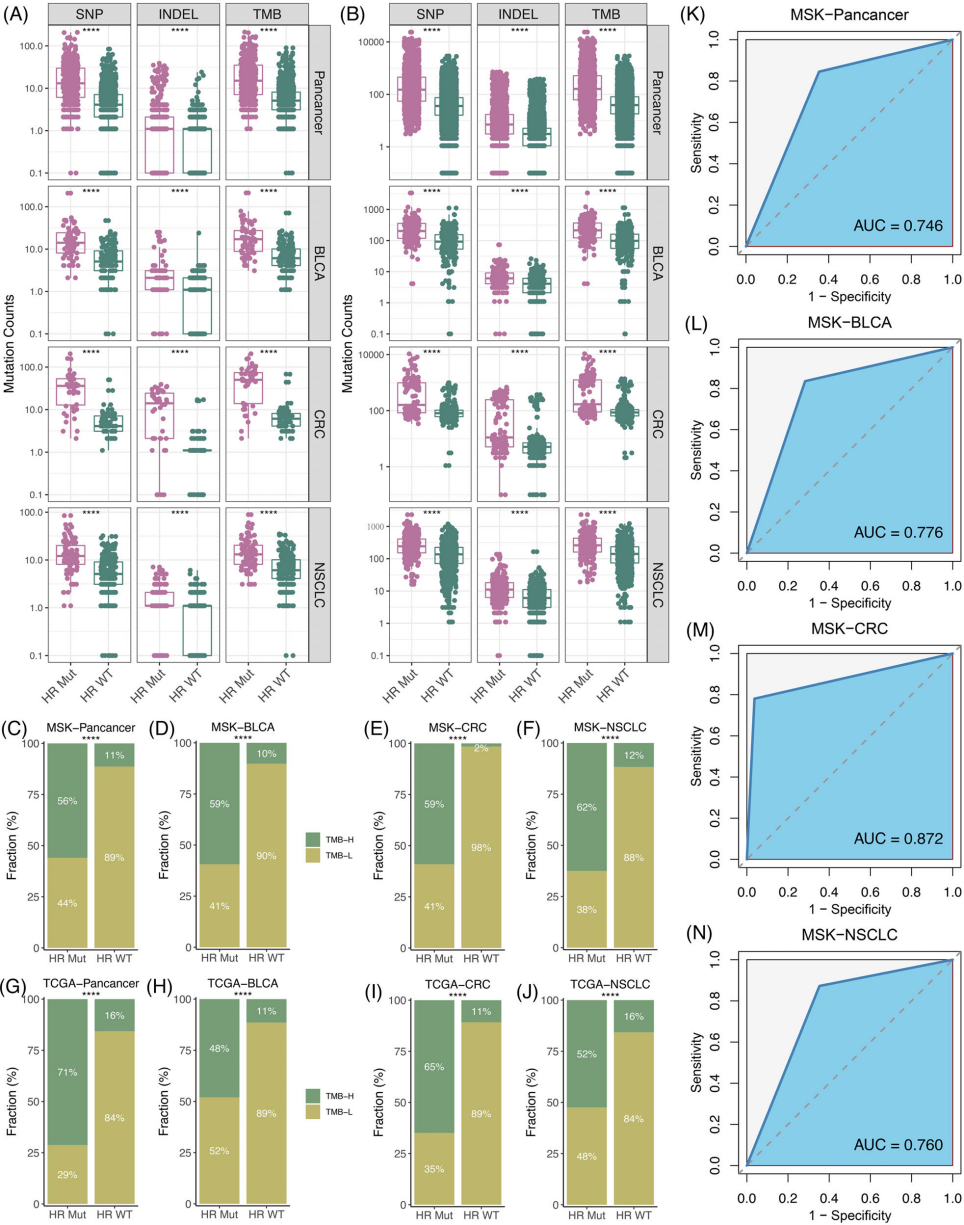
The association between homologous recombination (HR) mutations and tumour mutation burden (TMB). (A) Comparison of single nucleotide polymorphism (SNP), insertion and deletion (INDEL) and TMB between the HR‐Mut and HR‐WT phenotypes in MSK‐pan‐cancer, MSK‐bladder cancer (BLCA), MSK‐colorectal cancer (CRC) and MSK‐non‐small‐cell lung cancer (NSCLC). (B) Comparison of SNP, INDEL and TMB between the HR‐Mut and HR‐WT phenotypes in TCGA‐pan‐cancer, TCGA‐BLCA, TCGA‐CRC and TCGA‐NSCLC. (C‐J) The proportion of TMB‐high and TMB‐low in the HR‐Mut and HR‐WT phenotypes in MSK‐pan‐cancer (C), MSK‐BLCA (D), MSK‐CRC (E), MSK‐NSCLC (F), TCGA‐pan‐cancer (G), TCGA‐BLCA (H), TCGA‐CRC (I) and TCGA‐NSCLC (J). (K‐N) ROC curves of HR mutations to predict higher TMB in MSK‐pan‐cancer (K), MSK‐BLCA (L), MSK‐CRC (M) and MSK‐NSCLC (N). ****P < 0.0001

Subsequently, gene set enrichement analysis (GSEA) was applied to decipher the underlying mechanisms in terms of cancer Hallmarks, and 22 pathways dramatically enriched (Figure 4A). The HR‐Mut subtype was mainly correlated with cell cycle and interferon response, while the HR‐WT subtype enriched plenty of stromal activation‐related terms (Figure 4A). To further explore the tumour microenvironment (TME), the “ESTIMATE” R package was employed to infer the fraction of immune and stromal components.^8^ The HR‐Mut phenotype scored better in immune score and displayed lower stromal score and tumour purity (Figure S6A). There was no significant difference in the correlations between immune and stromal scores in the two HR phenotypes, and the tumour purity was lower as the immune and stromal scores were increasing in both subgroups (Figure 4B).

**FIGURE 4:**
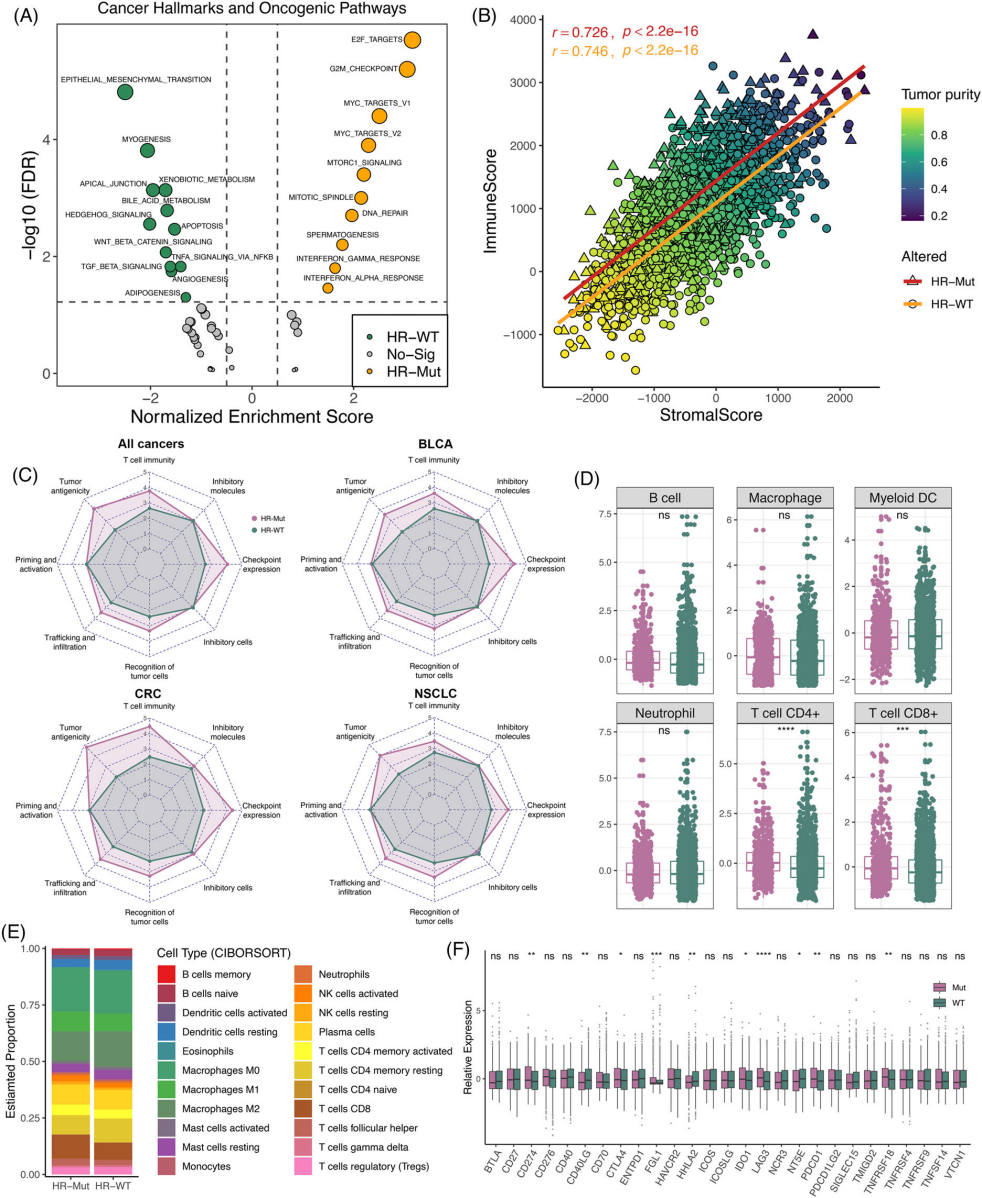
Tumour microenvironment (TME) and immunity characterization of the homologous recombination (HR) phenotypes. (A) GSEA analysis of cancer Hallmarks between the HR‐Mut and HR‐WT phenotypes. Green points represent pathways significantly related to HR‐Mut tumours, orange points represent pathways significantly related to HR‐WT tumours, and grey points represent pathways with no significance between the two phenotypes. (B) Scatterplots between stromal and immune scores with tumour purity gradient were shown, and its correlation coefficient was indicated by each phenotype. The colour grading corresponds to the tumour purity, indexed as shown on the colour bar at the top right of the panel. (C) Radar plots showed that the immunogram patterns of the two phenotypes. The axes of the radar chart were generated with the median immunogram score for the HR‐Mut and HR‐WT phenotypes, respectively. (D) The TIMER algorithm showed the infiltration difference of six immune cell populations including B, CD4+ T, CD8+ T, neutrophil, macrophage and myeloid dendritic cells between two phenotypes. (E) The CIBORSORT algorithm showed the proportion of 22 immune cells in the HR‐Mut and HR‐WT phenotypes. (F) Differences in the distribution of 27 immune checkpoint molecules between two phenotypes. ^ns^ *p* > 0.05; *p < 0.05; **p < 0.01; ***p < 0.001; ****p < 0.0001

In cancer‐immunity cycle,^9^ we found four key immune‐active steps including T cell immunity, tumour antigenicity, trafficking and infiltration, and recognition of tumour cells were significantly enhanced in the HR‐Mut phenotype. Meanwhile, the HR‐Mut phenotype also displayed the substantial enhancement of checkpoint expression, implying an immune‐hot but suppressive microenvironment (Figure 4C and Figure S6B). To gain more detailed insights into this issue, we applied TIMER to quantify the infiltration abundance of different immune cell populations.^10^ The infiltration abundance of CD4+ and CD8+ T cells was higher in HR‐Mut tumours, which further demonstrated T cell enrichment was a dominant factor in the TME of HR‐Mut tumours (Figure 4D). Meanwhile, the deconvolution algorithm CIBERSORT validated these results, and presented that the HR‐Mut phenotype had superior infiltration of activated nature killer cells, while the HR‐WT phenotype harbored more resting cells such as resting dendritic cells and resting CD4+ T memory cells (Figure 4E and Figure S6C). In addition, we observed the expression level of *CD274*, *PDCD1*, and *CTLA4* was superior in the HR‐Mut subtype, which may explain its better response to ICI treatment (Figure 4F and Figure S6B). There were also some other molecules that were significantly up‐regulated in HR‐Mut tumours, such as *FGL1*, *IDO1*, *LAG3* and *TNFRSF18*. Meanwhile, *CD40LG, HHLA2* and *NT5E* were highly expressed in HR‐WT tumours, which may be the potential targets to improve the prognosis of patients with the HR‐WT phenotype.

In conclusion, HR mutations are predictive of improved clinical outcomes in BLCA, CRC and NSCLC treated with ICI instead of non‐ICI. HR mutations could accurately predict TMB‐high, NAL‐high and MSI‐H, suggesting that HR mutations may be a promising surrogate for TMB, NAL and MSI estimation. HR mutations are also associated with the TME, immunity characteristics and immune checkpoints profiles. Our findings lay a foundation for the clinical application of HR mutations in cancer immunotherapy.

## CONFLICT OF INTEREST

The authors declare that there is no conflict of interest that could be perceived as prejudicing the impartiality of the research reported.

## Supporting information

Figure

Supporting information

Table
