# Supplementary material for: Somatic mutations in homologous recombination pathway predict favourable prognosis after immunotherapy across multiple cancer types
Source: Clin Transl Med. 2021 Dec 19;11(12):e619. doi: 10.1002/ctm2.619 (PMC8684773; doi:10.1002/ctm2.619)
Supplement: Supplementary file 1 — Figure [file CTM2-11-e619-s001.docx]

Supplementary Figure

- Figure S1
- Figure S2
- Figure S3
- Figure S4
- Figure S5
- Figure S6
- Figure S7


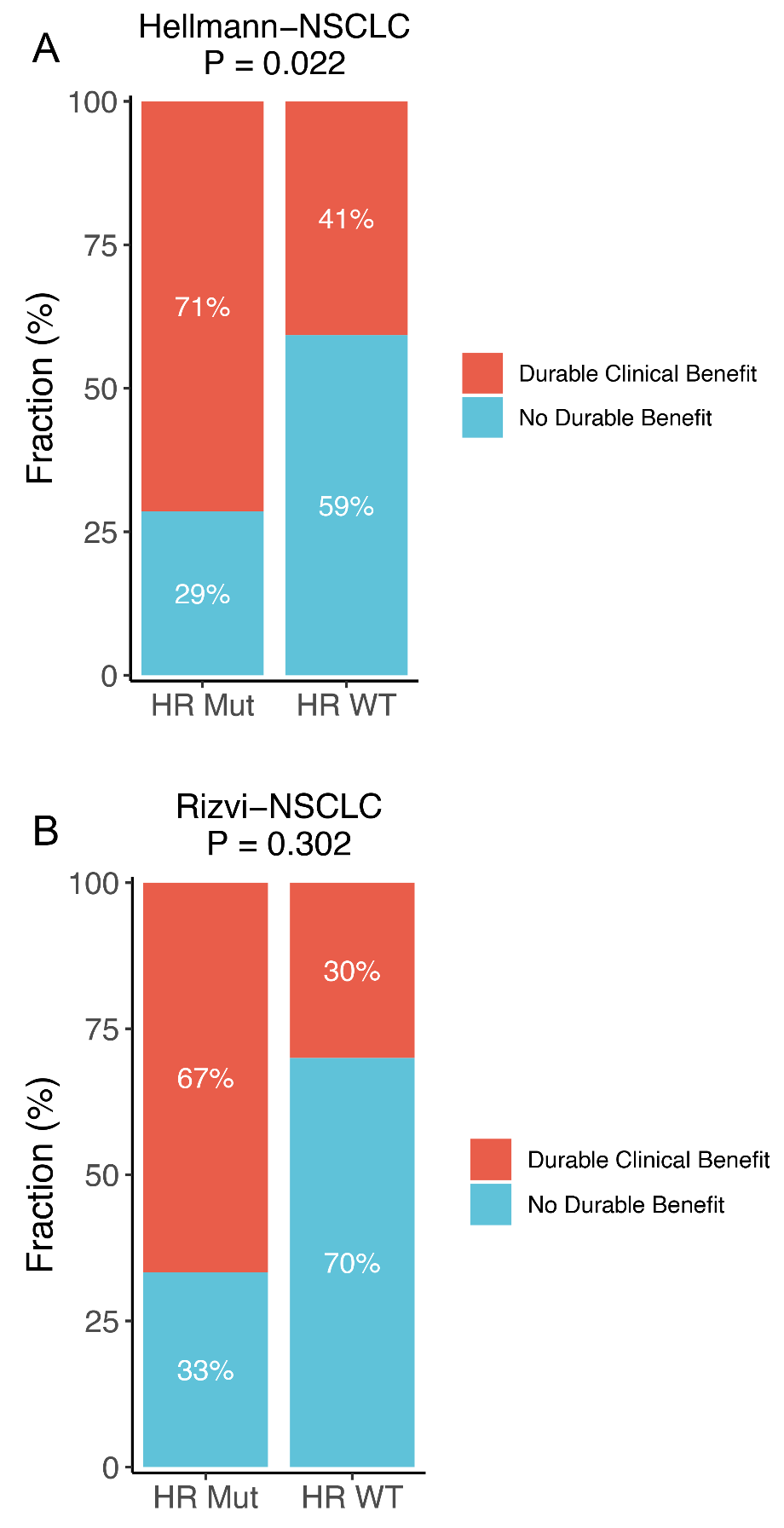


**Figure S1. The proportion of DCB in the HR-Mut and HR-WT phenotypes in Hellmann-NSCLC and Rizvi-NSCLC cohorts.**


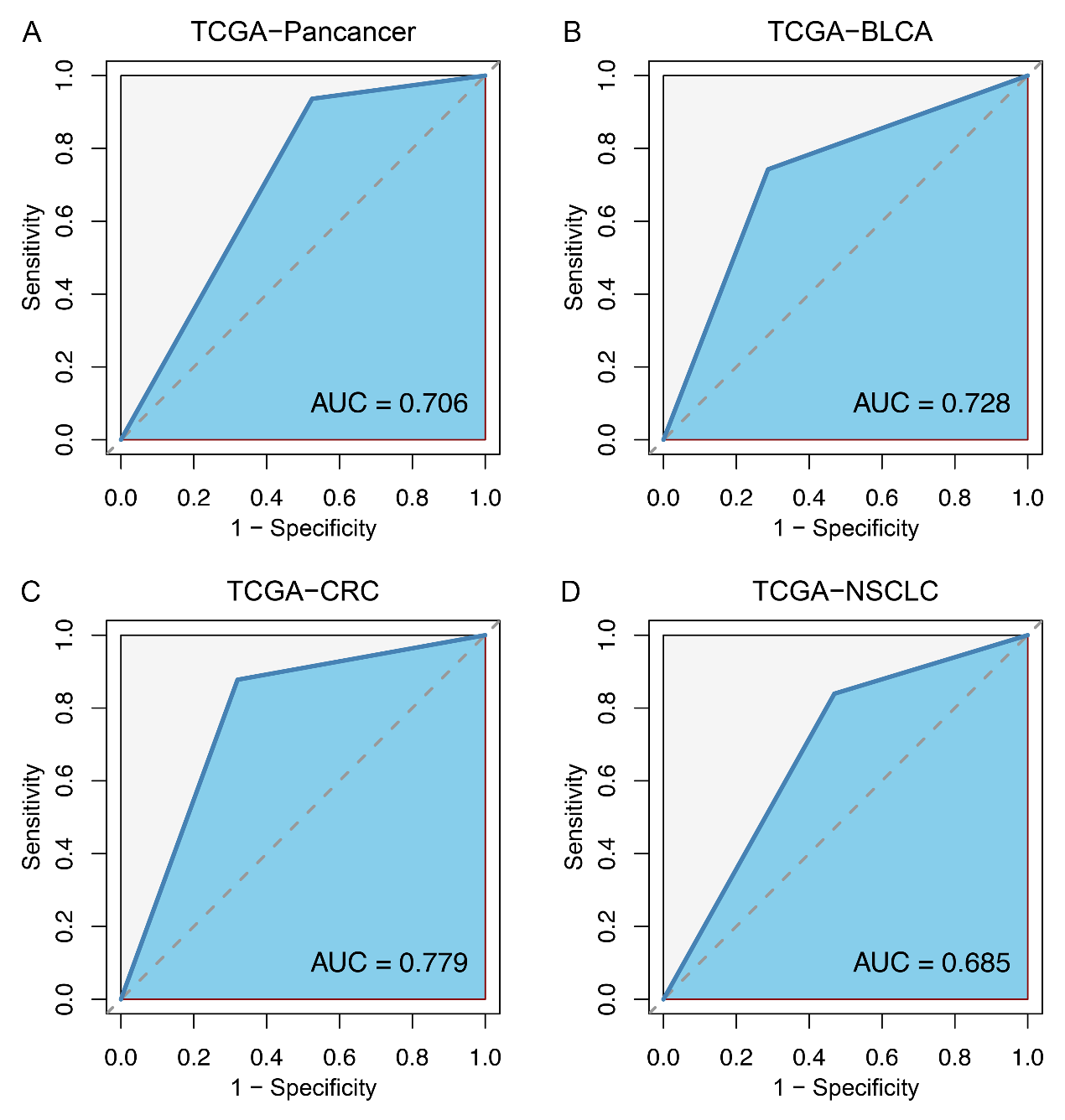


**Figure S2. ROC curves of HR mutations to predict higher TMB in TCGA-pan-cancer, TCGA-BLCA, TCGA-CRC, and TCGA-NSCLC.**


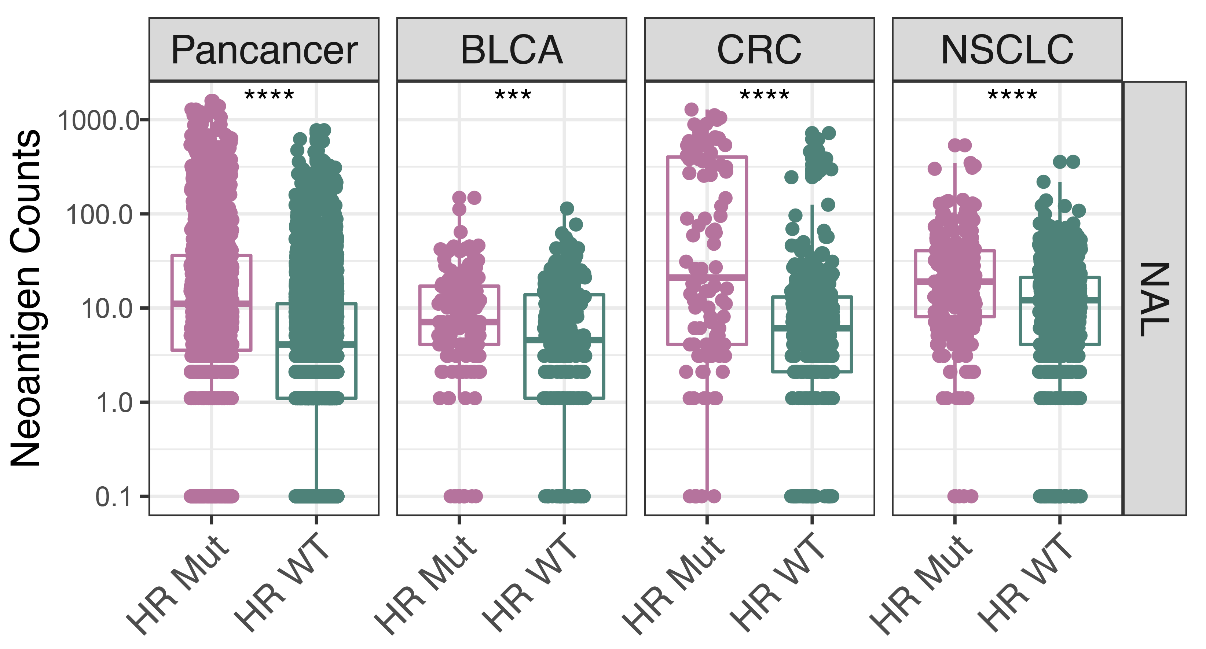


**Figure S3.** Comparison of NAL between the HR-Mut and HR-WT phenotypes in TCGA-pan-cancer, TCGA-BLCA, TCGA-CRC, and TCGA-NSCLC.


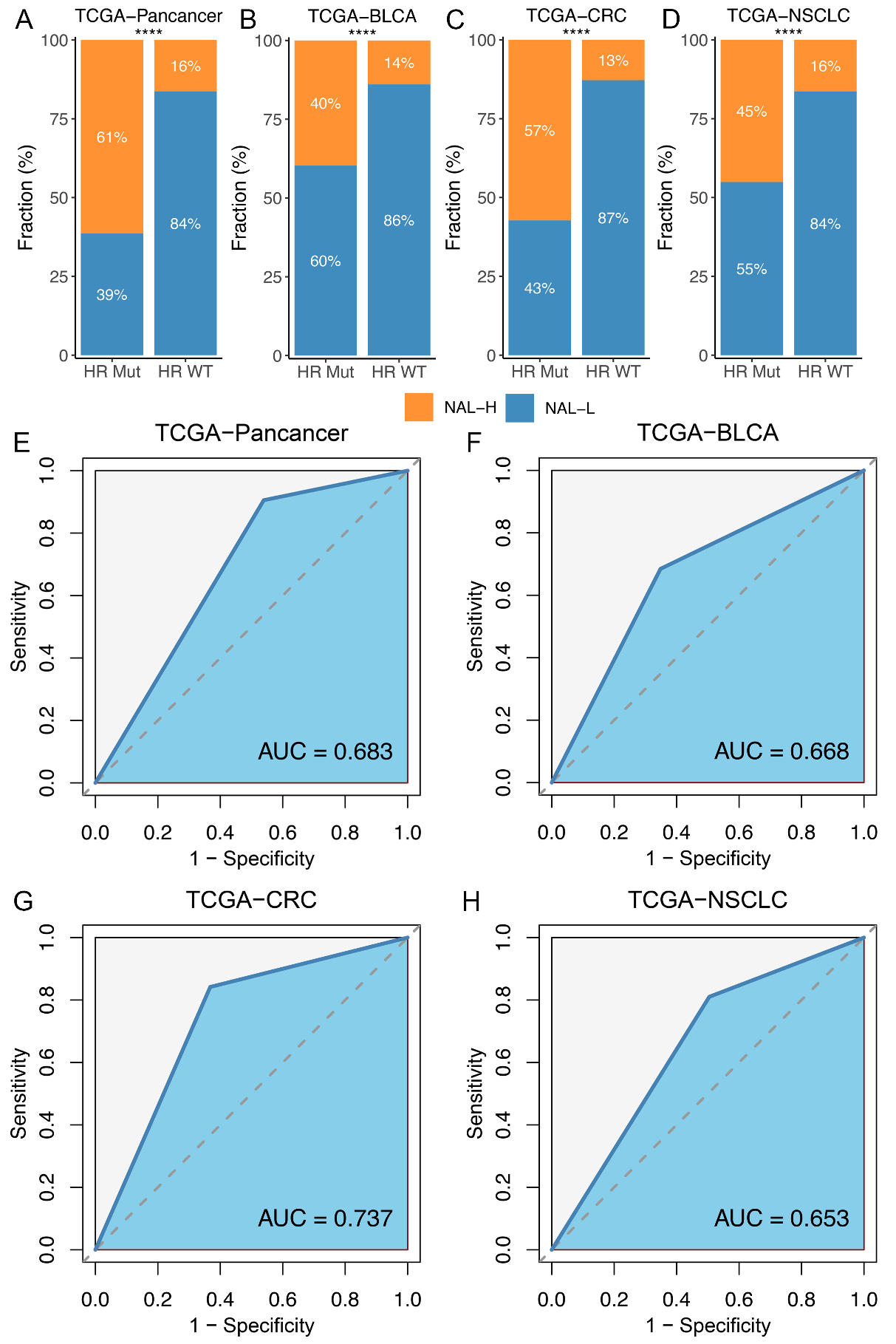


**Figure S4. The association between HR mutations and NAL. A-D.** The proportion of NAL-high and NAL-low in the HR-Mut and HR-WT phenotypes in TCGA-pan-cancer (**A**), TCGA-BLCA (**B**), TCGA-CRC (**C**), and TCGA-NSCLC (**D**). **E-H.** ROC curves of HR mutations to predict higher NAL in TCGA-pan-cancer (**E**), TCGA-BLCA (**F**), TCGA-CRC (**G**), and TCGA-NSCLC (**H**). *****P* <0.0001.


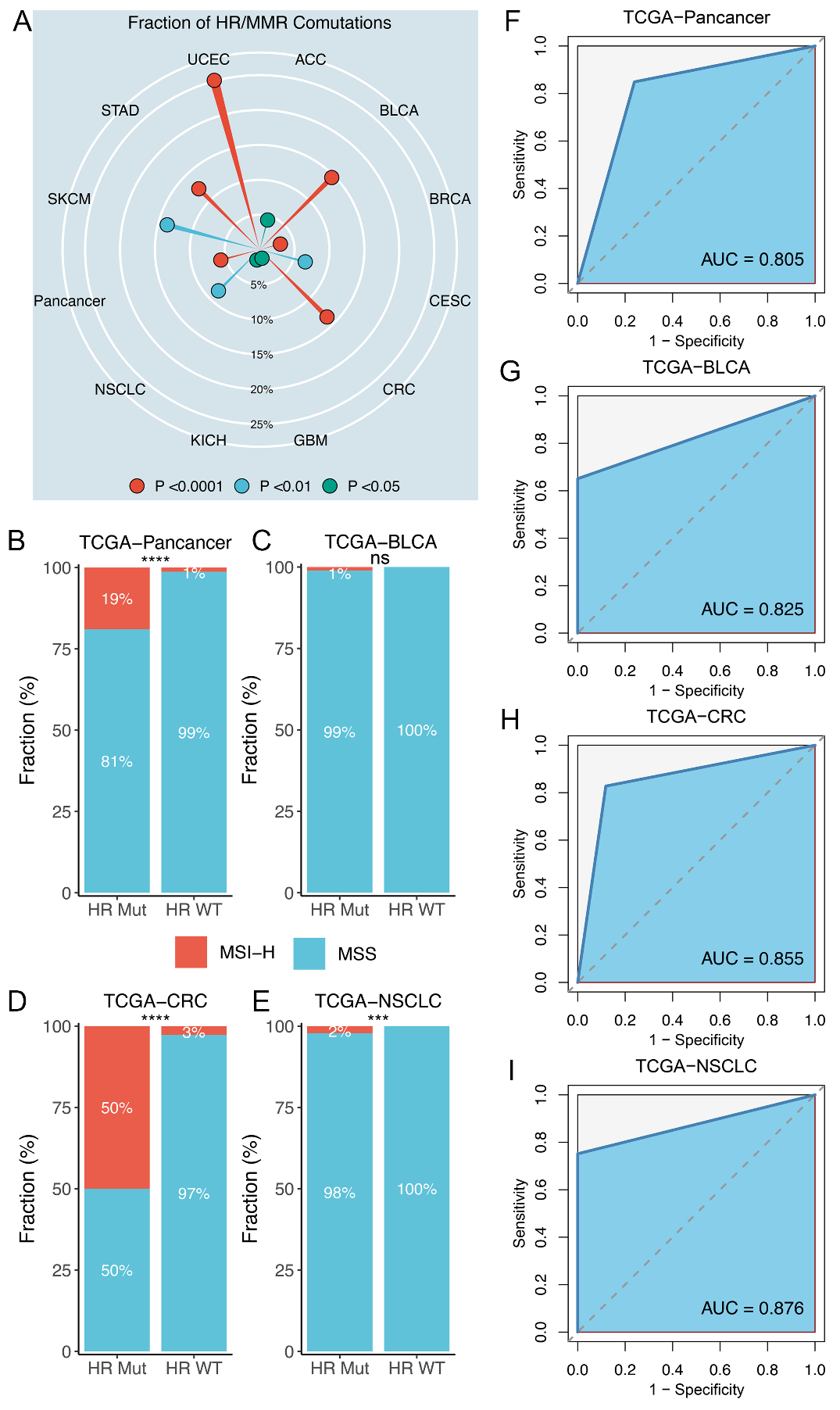


**Figure S5. The association between HR mutations and MSI. A.** Fraction of HR/MMR comutations in multiple cancer types. **B-E.** The proportion of MSI-H and MSS in the HR-Mut and HR-WT phenotypes in TCGA-pan-cancer (**B**), TCGA-BLCA (**C**), TCGA-CRC (**D**), and TCGA-NSCLC (**E**). **F-I.** ROC curves of HR mutations to predict MSI-H in TCGA-pan-cancer (**F**), TCGA-BLCA (**G**), TCGA-CRC (**H**), and TCGA-NSCLC (**I**). ^ns^*P* >0.05, ****P* <0.001, *****P* <0.0001.


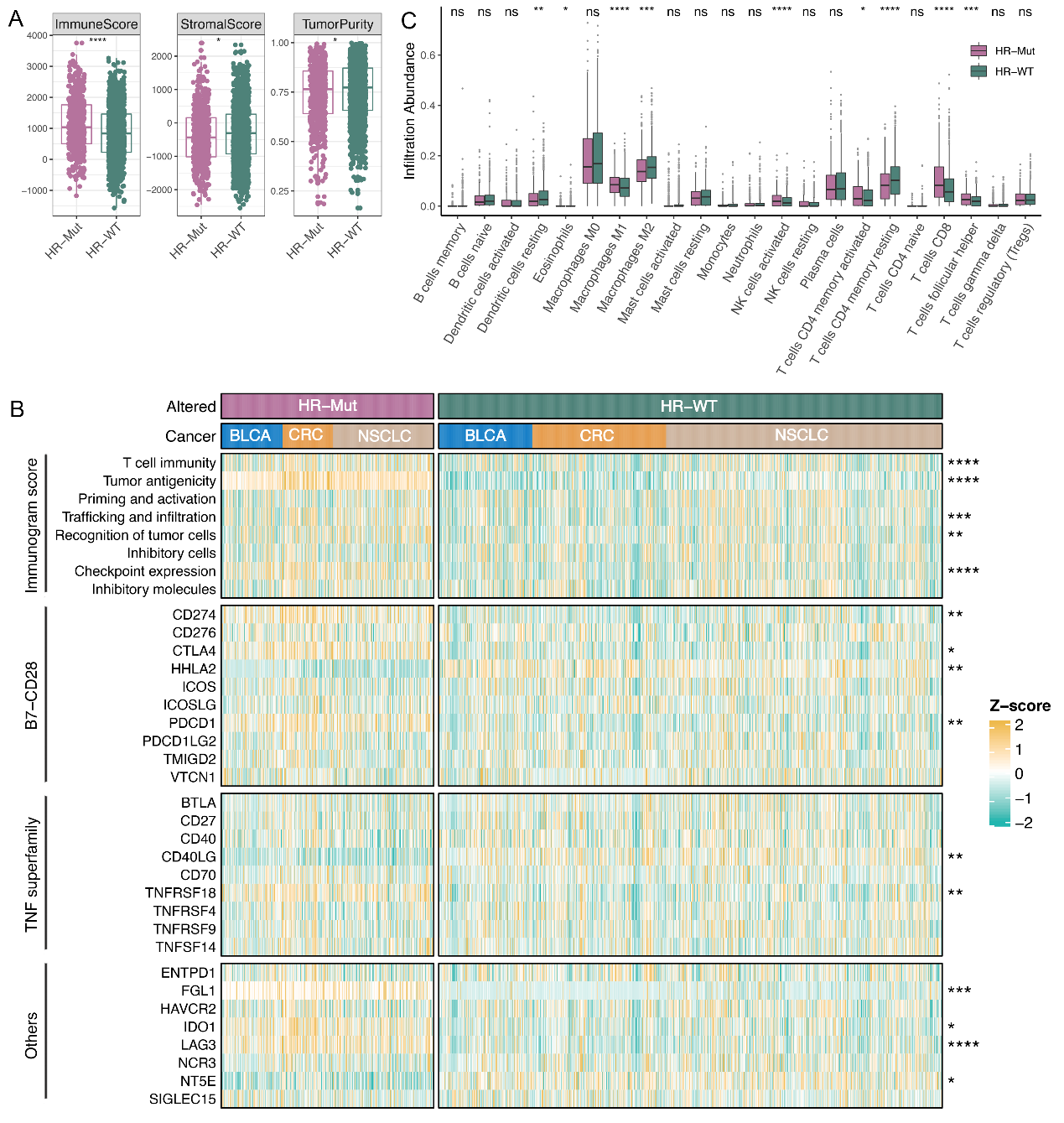


**Figure S6. TME and immunity characterization of the HR phenotypes. A.** Comparison of immune, stromal score, and tumor purity between the HR-Mut and HR-WT phenotypes. **B.** Heatmap of 8 immunogram scores and 27 immune checkpoint molecules between the HR-Mut and HR-WT phenotypes. C. Differences in the distribution of 22 immune cells infiltration between two phenotypes. ^ns^*P* >0.05, ^*^*P* <0.05, ^**^*P* <0.01, ^***^*P* <0.001, ^****^*P* <0.0001.


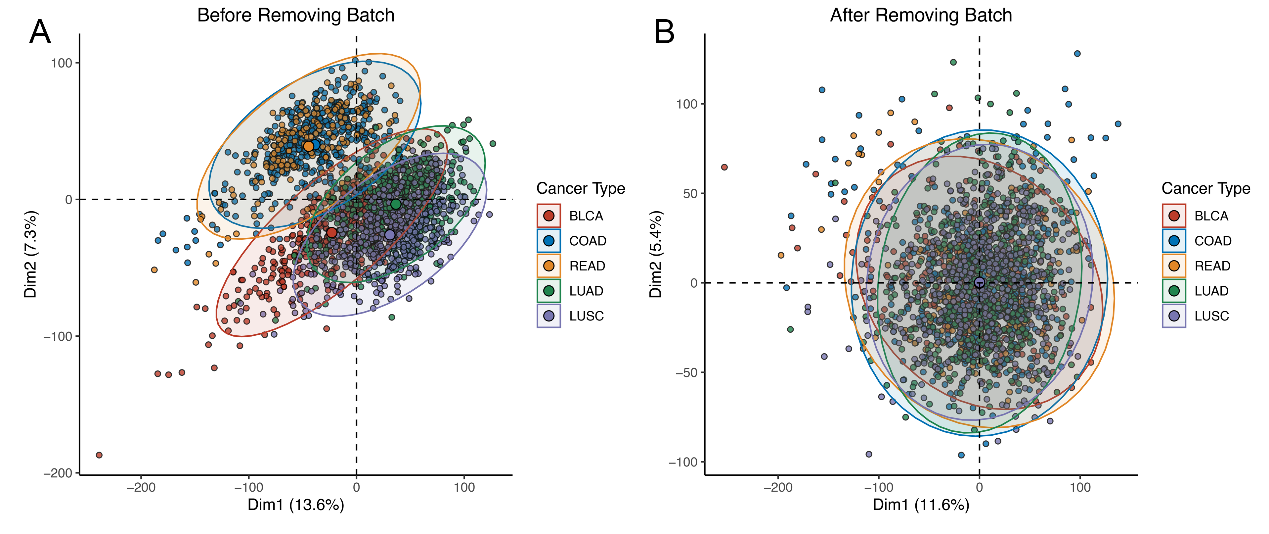


**Figure S7. Principal component analysis of transcriptome expression profiles before and after batch effect removal.**
