# Supplementary material for: Somatic mutations in homologous recombination pathway predict favourable prognosis after immunotherapy across multiple cancer types
Source: Clin Transl Med. 2021 Dec 19;11(12):e619. doi: 10.1002/ctm2.619 (PMC8684773; doi:10.1002/ctm2.619)
Supplement: Supplementary file 2 — Supporting information [file CTM2-11-e619-s002.docx]

**Supplementary method**

**Data sources**

A total of 1,752 cancer patients treated with ICI and 4,605 non-ICI-treated patients were enrolled. ICI-treated patients enrolled in this study had received at least one dose of immunotherapy (atezolizumab, avelumab, durvalumab, ipilimumab, nivolumab, pembrolizumab or tremelimumab). Table S1 summarized the data sources and details of this study.

***MSK ICI cohort*.** The MSK ICI cohort contained 1,661 patients in 10 cancer types, who received at least one dose of ICI therapy^1^. The clinical information and DNA sequencing data (MSK-IMPACT sequencing) were retrieved from the cBioPortal ([www.cbioportal.org](http://www.cbioportal.org)). The following cancer types were included: CRC (n =110), melanoma (n =320), bladder cancer (BLCA, n =215), NSCLC (n =350), renal cell carcinoma (n =151), esophagogastric cancer (n =126), head and neck cancer (HNCA, n =139), breast cancer (n =44), glioma (n =177), cancer of unknown primary (CUP, n=88). The endpoint event was the overall survival (OS) of patients after receiving ICI treatment.

***NSCLC ICI cohort*.** Data from a total of 91 NSCLC patients was derived from the following studies: Hellmann and colleagues^2^ (n =75) and Rizvi and colleagues^3^ (n =16). Patients in Hellmann cohort were treated with *PD-1* plus *CTLA-4* blockade, and patients in Rizvi cohort were treated with *PD-1* blockade. All patients were performed with whole-exome sequencing. The endpoint events were the durable benefit and progression-free survival (PFS) of patients after receiving ICI treatment. The durable clinical benefit (DCB) was defined as partial or stable response lasting >6 months.

***Non-ICI cohorts for BLCA, CRC, and NSCLC*.** We enrolled a total of 11 non-ICI cohorts, as follows, three BLCA cohorts including TCGA-BLCA (n =404), Hikmat-BLCA^4^ (n =30), and Sfakianos-BLCA^5^ (n =77), three CRC cohorts including TCGA-CRC (n =501), ICGC-COCA-CN (n =309), and Yaeger-CRC^6^ (n =1134), and five NSCLC cohorts including TCGA-NSCLC (TCGA-LUAD and TCGA-LUSC, n =971), MSKCC-2020-LUAD (n =604), ICGC-LUSC-KR (n =170), Chen-LUAD^7^ (n =305), and PDX-NSCLC (n =100). All patients did not receive ICI treatment and have both clinical information and DNA sequencing data. The endpoint event was the OS.

***Multi-omics data for*** ***B******LCA, CRC, and NSCLC*.** The multi-omics data for BLCA, CRC, and NSCLC, including HumanMethylation450 array, copy number alteration (CNA) data, whole-exome sequencing data, and transcriptome profiling data, were derived from TCGA portal (<https://portal.gdc.cancer.gov>).

***Assessments of TMB, NAL, and MSI***

TMB was defined as the total non-silent somatic mutation counts in coding regions, encompassing missense, nonsense, frame shift insertion, frame shift deletion, in-frame insertion, in-frame deletion, and splice site mutation. Data in the MSK ICI cohort was directly retrieved from cBioPortal online, which was processed via MSK-IMPACT. The TCGA pan-cancer cohort contained 10,114 patients across 32 cancer types, and the whole-exome sequencing data was obtained from TCGA portal. The “maftools” R package was utilized to process mutation data and calculate the TMB of each patient. Neoantigens of 5,935 solid tumors measured by TCGA official were available in TCIA database (<https://tcia.at/neoantigens>)^8^. TMB or NAL was categorized into high and low groups according to the top quartile^9^. The MSI of 5,930 solid tumors across 18 tumor types examined by Hause and colleagues was also collected^10^.

**Mutational status of HR genes**

The “maftools” software was employed to process the DNA sequencing data. Based on the recommend of Memorial Sloan Kettering Cancer Center (MSKCC), HR mutations were defined as any non-silent mutations in 17 recommended genes, including *ATM*, *BAP1*, *BARD1*, *BLM*, *BRCA1*, *BRCA2*, *BRIP1*, *CHEK2*, *ABRAXAS1*, *FANCA*, *FANCC*, *NBN*, *PALB2*, *RAD50*, *RAD51*, *RAD51C*, and *RTEL1*^11, 12^. According to the presence or absence of mutations in those genes, patients were segmented into HR mutation (HR-Mut) and HR wild-type (HR-WT) subgroups.

**Gene set enrichment analysis**

To reveal the potential molecular mechanisms underlying the HR phenotypes, the gene set enrichment analysis (GSEA) algorithm implemented in “clusterProfiler” R package was performed to identify dramatically enriched terms related to cancer Hallmark pathways (h.all.v7.2.symbols.gmt). Permutations were set to 1000 to obtain a normalized enrichment score (NES). Gene sets with false discovery rate (FDR) <0.05 were considered to be significantly enriched.

**TME** **characterization analysis**

Gene expression profiles were utilized to decipher the TME characterization of tumor samples with multiple bioinformatics tools. The “ESTIMATE” R package was utilized to infer the fraction of stromal and immune fraction in solid tumor samples, and generated immune, stromal scores, and tumor purity. Based on the rationale that immunity within tumors is a dynamic process, Karasaki and colleagues^13^ have proposed an immunogram for the cancer-immunity cycle (CIC) depicted by eight axes of the immunogram score (IGS): IGS1, T cell immunity; IGS2, tumor antigenicity; IGS3, priming and activation (actived dendric cell enrichment); IGS4, trafficking and infiltration; IGS5, recognition of tumor cells; IGS6, inhibitory cells; IGS7, checkpoint expression; and IGS8, inhibitory molecules. The gene sets of IGS1-IGS8 were retrieved from a previous study^13^. The single‐sample gene‐set enrichment analysis (ssGSEA) approach was leveraged to measure the IGS, and the immunogram radar displayed the mean IGS value of two HR phenotypes. To further evaluate the infiltration abundance of immune cell populations in tumor tissues, we applied two different tools, including TIMER and CIBERSORT algorithms.

**Delineate the immune checkpoints profiles**

To obtain the normalized gene expression value, the “DESeq2” R package was employed to performed variance stabilized transformations on the raw read counts (HTseq) from TCGA database. The “ComBat” algorithm was used to reduce the likelihood of batch effects from non-biological technical biases (Figure S7). Next, we compared the expression differences of 27 immune checkpoint members, including B7-CD28 family (*PD-L1*, *PD-L2*, *PD-1*, *CDLA4*, *CD276*, *HHLA2*, *ICOS*, *ICOSLG*, *TMIGD2*, and *VTCN1*)^14^, the TNF superfamily (*BTLA*, *CD27*, *CD40*, *CD40LG*, *CD70*, *TNFRSF18*, *TNFRSF4*, *TNFRSF9*, and *TNFSF14*)^15^, and several other molecules (*ENTPD1*, *FGL1*, *HAVCR2*, *IDO1*, *LAG3*, *NCR3*, *NT5E*, and *SIGLEC15*)^16, 17^.

**Statistical analysis**

All data processing, statistical analysis, and plotting were performed in R 4.0.5 software. The Kaplan-Meier method and the log-rank test were utilized to estimate the different survival between two groups. The “survival” R package was utilized to perform Cox regression analysis. Fisher’s exact test or Pearson's chi-squared test was applied to compare categorical variables. Continuous variables were compared between two groups through the Wilcoxon rank-sum test or T test. Correlations between two continuous variables were assessed via Pearson’s correlation coefficients. The receiver operating characteristic curve (ROC) was implemented using the “pROC” R package. All statistical tests were two-sided. *P* <0.05 was regarded as statistically significant.

**Reference**

1. Samstein RM, Lee CH, Shoushtari AN, et al. Tumor mutational load predicts survival after immunotherapy across multiple cancer types. *Nat Genet*. 2019; 51(2):202-206.

2. Hellmann MD, Nathanson T, Rizvi H, et al. Genomic Features of Response to Combination Immunotherapy in Patients with Advanced Non-Small-Cell Lung Cancer. *Cancer Cell*. 2018; 33(5):843-852 e844.

3. Rizvi NA, Hellmann MD, Snyder A, et al. Cancer immunology. Mutational landscape determines sensitivity to PD-1 blockade in non-small cell lung cancer. *Science*. 2015; 348(6230):124-128.

4. Al-Ahmadie HA, Iyer G, Lee BH, et al. Frequent somatic CDH1 loss-of-function mutations in plasmacytoid variant bladder cancer. *Nat Genet*. 2016; 48(4):356-358.

5. Kim PH, Cha EK, Sfakianos JP, et al. Genomic predictors of survival in patients with high-grade urothelial carcinoma of the bladder. *Eur Urol*. 2015; 67(2):198-201.

6. Yaeger R, Chatila WK, Lipsyc MD, et al. Clinical Sequencing Defines the Genomic Landscape of Metastatic Colorectal Cancer. *Cancer Cell*. 2018; 33(1):125-136 e123.

7. Chen J, Yang H, Teo ASM, et al. Genomic landscape of lung adenocarcinoma in East Asians. *Nat Genet*. 2020; 52(2):177-186.

8. Charoentong P, Finotello F, Angelova M, et al. Pan-cancer Immunogenomic Analyses Reveal Genotype-Immunophenotype Relationships and Predictors of Response to Checkpoint Blockade. *Cell Rep*. 2017; 18(1):248-262.

9. Wang Z, Zhao J, Wang G, et al. Comutations in DNA Damage Response Pathways Serve as Potential Biomarkers for Immune Checkpoint Blockade. *Cancer Res*. 2018; 78(22):6486-6496.

10. Hause RJ, Pritchard CC, Shendure J, Salipante SJ. Classification and characterization of microsatellite instability across 18 cancer types. *Nat Med*. 2016; 22(11):1342-1350.

11. Park W, Chen J, Chou JF, et al. Genomic Methods Identify Homologous Recombination Deficiency in Pancreas Adenocarcinoma and Optimize Treatment Selection. *Clin Cancer Res*. 2020; 26(13):3239-3247.

12. Lord CJ, Ashworth A. BRCAness revisited. *Nat Rev Cancer*. 2016; 16(2):110-120.

13. Karasaki T, Nagayama K, Kuwano H, et al. An Immunogram for the Cancer-Immunity Cycle: Towards Personalized Immunotherapy of Lung Cancer. *J Thorac Oncol*. 2017; 12(5):791-803.

14. Janakiram M, Chinai JM, Zhao A, Sparano JA, Zang X. HHLA2 and TMIGD2: new immunotherapeutic targets of the B7 and CD28 families. *Oncoimmunology*. 2015; 4(8):e1026534.

15. Ward-Kavanagh LK, Lin WW, Sedy JR, Ware CF. The TNF Receptor Superfamily in Co-stimulating and Co-inhibitory Responses. *Immunity*. 2016; 44(5):1005-1019.

16. Chretien S, Zerdes I, Bergh J, Matikas A, Foukakis T. Beyond PD-1/PD-L1 Inhibition: What the Future Holds for Breast Cancer Immunotherapy. *Cancers (Basel)*. 2019; 11(5).

17. Wang J, Sanmamed MF, Datar I, et al. Fibrinogen-like Protein 1 Is a Major Immune Inhibitory Ligand of LAG-3. *Cell*. 2019; 176(1-2):334-347 e312.
